# (-)-Pentazocine induces visceral chemical antinociception, but not thermal, mechanical, or somatic chemical antinociception, in μ-opioid receptor knockout mice

**DOI:** 10.1186/1744-8069-7-23

**Published:** 2011-04-10

**Authors:** Soichiro Ide, Masabumi Minami, George R Uhl, Masamichi Satoh, Ichiro Sora, Kazutaka Ikeda

**Affiliations:** 1 Department of Pharmacology, Graduate School of Pharmaceutical Sciences, Hokkaido University, Sapporo 060-0812, Japan; 2 Research Project for Addictive Substances, Tokyo Metropolitan Institute of Medical Science, Tokyo 156-8506, Japan; 3 Molecular Neurobiology, National Institute on Drug Abuse, Baltimore, Maryland 21224, USA; 4 Department of Pharmacy, Yasuda Women's University, Hiroshima 731-0153, Japan; 5 Division of Psychobiology, Department of Neuroscience, Tohoku University Graduate School of Medicine, Sendai 980-8574, Japan

**Keywords:** Opioid receptor, Knockout mice, Pentazocine, Antinociception

## Abstract

**Background:**

(-)-Pentazocine has been hypothesized to induce analgesia via the κ-opioid (KOP) receptor, although the involvement of other opioid receptor subtypes in the effects of pentazocine remains unknown. In this study, we investigated the role of the μ-opioid (MOP) receptor in thermal, mechanical, and chemical antinociception induced by (-)-pentazocine using MOP receptor knockout (MOP-KO) mice.

**Results:**

(-)-Pentazocine-induced thermal antinociception, assessed by the hot-plate and tail-flick tests, was significantly reduced in heterozygous and abolished in homozygous MOP-KO mice compared with wildtype mice. The results obtained from the (-)-pentazocine-induced mechanical and somatic chemical antinociception experiments, which used the hind-paw pressure and formalin tests, were similar to the results obtained from the thermal antinociception experiments in these mice. However, (-)-pentazocine retained its ability to induce significant visceral chemical antinociception, assessed by the writhing test, in homozygous MOP-KO mice, an effect that was completely blocked by pretreatment with nor-binaltorphimine, a KOP receptor antagonist. *In vitro *binding and cyclic adenosine monophosphate assays showed that (-)-pentazocine possessed higher affinity for KOP and MOP receptors than for δ-opioid receptors.

**Conclusions:**

The present study demonstrated the abolition of the thermal, mechanical, and somatic chemical antinociceptive effects of (-)-pentazocine and retention of the visceral chemical antinociceptive effects of (-)-pentazocine in MOP-KO mice. These results suggest that the MOP receptor plays a pivotal role in thermal, mechanical, and somatic chemical antinociception induced by (-)-pentazocine, whereas the KOP receptor is involved in visceral chemical antinociception induced by (-)-pentazocine.

## Background

The racemic compound (±)-pentazocine is used for the management of mild to moderate pain in humans. (-)-Pentazocine is known to act as an opioid analgesic, and (+)-pentazocine is a σ receptor agonist without analgesic effects. The antinociceptive effects of (-)-pentazocine are reportedly mediated by its agonist action at the κ-opioid (KOP) receptor [1]. A previous report showed that the antinociceptive effects of (-)-pentazocine were antagonized by nor-binaltorphimine (nor-BNI, a selective KOP receptor antagonist) but not by β-funaltrexamine (a selective μ-opioid [MOP] receptor antagonist) in the mouse tail-flick test [2]. However, (-)-pentazocine reportedly binds not only KOP receptors but also MOP receptors with high affinity [2] and acts as a MOP receptor partial agonist. Furthermore, the antinociceptive effects of (-)-pentazocine were antagonized by β-funaltrexamine in the mouse hot-plate test [3] and writhing test [4]. Thus, the role of the MOP receptor in the antinociceptive effects of (-)-pentazocine remains unclear. Moreover, the most selective ligands for specific opioid receptor subtypes (e.g., β-funaltrexamine for the MOP receptor, naltrindole for the δ-opioid [DOP] receptor, and nor-BNI for the KOP receptor) possess certain affinities for other opioid receptor subtypes [5]. Thus, the precise molecular mechanisms that underlie the antinociceptive effects of (-)-pentazocine have not been clearly delineated by traditional pharmacological studies that use only selective ligands.

Developing mice that lack the MOP receptor gene has made possible the discovery of the molecular mechanisms that underlie the effects of opioids [6-9]. Both the analgesic and rewarding effects of morphine are abolished in MOP receptor knockout (MOP-KO) mice [7-9]. Buprenorphine, a nonselective opioid receptor partial agonist, exerts no analgesic effects in the tail-flick and hot-plate tests but a significant rewarding effect in the conditioned place preference paradigm in homozygous MOP-KO mice [10]. These observations are especially interesting because the distributions of DOP and KOP receptors are not apparently altered in MOP-KO mice [6,7,9]. Furthermore, butorphanol, a nonselective opioid receptor partial agonist, exerts no thermal or mechanical antinociceptive effects but exerts visceral chemical antinociceptive effects that are sensitive to nor-BNI in MOP-KO mice [11]. Although several compensatory changes might occur in KO animals, these animal models have potential utility in the investigation of the *in vivo *roles of specific proteins. Thus, the use of MOP-KO mice has provided novel theories on the molecular mechanisms that underlie the effects of opioid ligands. The present study investigated the molecular mechanisms that underlie the antinociceptive effects of (-)-pentazocine using various types of nociceptive stimuli in MOP-KO mice.

## Methods

### Animals

The present study used wildtype, heterozygous, and homozygous MOP-KO mouse littermates from heterozygous/heterozygous MOP-KO crosses on a C57BL/6J genetic background (backcrossed at least 10 generations) as previously described [8]. The experimental procedures and housing conditions were approved by the Institutional Animal Care and Use Committee, and all animal care and treatment were in accordance with our institutional animal experimentation guidelines. Naive adult (>10 weeks old) male and female mice were group housed in an animal facility maintained at 22 ± 2°C and 55 ± 5% relative humidity under a 12 h/12 h light/dark cycle with lights on at 8:00 AM and off at 8:00 PM. Food and water were available *ad libitum*.

### Drugs

(-)-Pentazocine and nor-BNI dihydrochloride were purchased from Sigma Chemical Co. (St. Louis, MO). For the *in vitro *assays, [D-Ala^2^,*N*-MePhe^4^,Gly-ol^5^]enkephalin (DAMGO), a MOP-selective agonist, and [D-Pen^2^,D-Pen^5^]enkephalin (DPDPE), a DOP agonist, were purchased from Peninsula Laboratories Ltd. (Merseyside, UK). (+)-(5α,7α,8β)-*N*-methyl-*N*-[7-(1-pyrrolidinyl)-1-oxaspirol[4,5]dec-8-yl]benzeneacetamide (U69593), a KOP-selective agonist, was a gift from Upjohn (Kalamazoo, MI). [tyrosyl-3,5-^3^H(N)]DAMGO (50.5 Ci/mmol), [phenyl-3,4-^3^H]U69593 (47.5 Ci/mmol), and [tyrosyl-2,6-^3^H(N)]DPDPE (33.0 Ci/mmol) were purchased from DuPont-New England Nuclear (Boston, MA).

### Antinociceptive tests

Thermal antinociception was evaluated using the hot-plate and tail-flick tests. Hot-plate testing was performed according to the method of Woolfe and MacDonald (1944) [12] with slight modifications. A commercially available apparatus that consisted of an acrylic resin cage (20 × 25 × 25 cm, width × length × height) and a temperature-controlled aluminum plate (Model MK-350A, Muromachi Kikai Co., Tokyo, Japan) was used for this test. Mice were placed on a 52 ± 0.2°C hot-plate, and the latencies to lick the hind-paw and jump were recorded. We selected a relatively low temperature (52°C) to examine the mild thermal antinociceptive effects of opioid partial agonists [10]. The cut-off time was 60 s. Tail-flick testing was performed according to the method of D'Amour and Smith (1941) [13] with slight modifications using a commercially available apparatus that consisted of an irradiator for heat stimulation and a photosensor for the detection of tail-flick behavior (Model MK-330A, Muromachi Kikai Co., Tokyo, Japan). The mice were loosely wrapped in a felt towel. Their tails were heated, and tail-flick latencies were automatically recorded. The cut-off time was 15 s. The tail-flick test was followed by the hot-plate test, and both tests were conducted in the same mice. Mechanical antinociception was evaluated using the hind-paw pressure test according to the method of Randall and Selitto (1957) [14] with slight modifications using a commercially available apparatus (Pressure Analgesy-Meter, Model MK-201D, Muromachi Kikai Co., Tokyo, Japan). The mice were loosely wrapped in a felt towel. Their hind-paws were gradually pressed, and hind-paw withdrawal and struggle latencies were automatically recorded. The cut-off pressure was 250 mmHg. The drug injection volume was 10 ml/kg. (-)-Pentazocine was administered at doses of 3, 7, 20, and 26 mg/kg (s.c.), for cumulative doses of 3, 10, 30, and 56 mg/kg, respectively. Tail-flick, hot-plate, and hind-paw pressure tests were conducted 20 min after each drug injection, and then the next dose of drug was injected immediately after these tests.

The hot-plate, tail-flick, and hind-paw pressure responses of each mouse in the drug-induced antinociception tests were converted to the percentage of maximal possible effect (%MPE) according to the following formula:

Visceral chemical antinociception was evaluated using the writhing test (Collier et al., 1968) [15]. Acetic acid (0.6% v/v, 10 ml/kg) was injected intraperitoneally (i.p.), and the mouse was placed in a large plastic cage. The intensity of nociceptive behavior was quantified by counting the total number of writhes that occurred between 0 and 15 min after the acetic acid injection. The writhing response consists of contraction of the abdominal muscles. Nociception is expressed as a writhing score during the 15 min period. (-)-Pentazocine (10 mg/kg, s.c.) or saline was administered 10 min before the acetic acid injection in a blind manner. nor-BNI (10 and 20 mg/kg, s.c.) was administered 24 h before the (-)-pentazocine injection.

Somatic chemical antinociception was evaluated using the formalin test in a blind manner as previously described [16]. Formalin (5% v/v, 20 μl) was injected into the right hind-paw (intraplantar), and the mouse was placed in a large plastic cage. The amount of time the mouse spent elevating, licking, shaking, or biting the injected paw was measured for each 5 min period during a 60 min session. Nociception was quantified using a rating scale by assigning weights to the following categories of nociceptive behavior: category 0 (weight is evenly distributed among all paws), category 1 (injected paw is lifted), category 2 (injected paw is licked, shaken, or bitten). The nociceptive score was calculated for each 5 min (300 s) period using the following formula:

(-)-Pentazocine (10 mg/kg, s.c.) or saline was administered 10 min before formalin injection (intraplantar) in a blind manner.

### Stable expression of human opioid receptors in Chinese hamster ovary cells

Chinese hamster ovary (CHO) cell lines that stably express human MOP, DOP, and KOP (MOP/CHO, DOP/CHO, and KOP/CHO, respectively) were established as previously described [10]. The *K*_*d *_values of [^3^H]DAMGO binding to MOP, [^3^H]DPDPE binding to DOP, and [^3^H]U69593 binding to KOP were 1.7 ± 0.3 nM (*n *= 4), 2.2 ± 0.2 nM (*n *= 4), and 2.5 ± 0.2 nM (*n *= 3), respectively. The *B*_*max *_estimates of receptor densities in these cell lines were 2300 ± 160, 3000 ± 270, and 5000 ± 450 fmol/mg protein, respectively.

### Radioligand binding assay

Binding assays were performed as previously described [17] with slight modifications. Expressing cells were harvested after 65 h in culture, homogenized in 50 mM Tris buffer (pH 7.4) that contained 10 mM MgCl_2 _and 1 mM EDTA, pelleted by centrifugation for 20 min at 30000 × *g*, and resuspended in the same buffer. For the saturation binding assays, cell membrane suspensions were incubated for 60 min at 25°C with various concentrations of [^3^H]DAMGO for the human MOP receptor, [^3^H]DPDPE for the human DOP receptor, or [^3^H]U69593 for the human KOP receptor. Nonspecific binding was determined in the presence of 10 mM unlabeled ligands. For the competitive binding assays, the cell membrane suspensions were incubated for 60 min at 25°C with 2 nM [^3^H]DAMGO for the human MOP receptor, 2 nM [^3^H]DPDPE for the human DOP receptor, or 3 nM [^3^H]U69593 for the human KOP receptor in the presence of various concentrations of ligands. After incubation for 60 min, the membrane suspensions were rapidly filtrated, and the radioactivity of each filter was then measured by liquid scintillation counting. The *K*_*d *_values of the radiolabeled ligands were obtained by Scatchard analysis of the data from the saturation binding assay. For the competitive binding assay, non-linear regression analysis using a one-competition model (GraphPad Prism, GraphPad, San Diego, CA) was conducted to estimate the inhibitory concentration at 50% (*IC*_*50*_). *K*_*i *_values were calculated from the *IC*_*50 *_values obtained from the competitive binding assay using the equation *K*_*i *_*= IC*_*50 *_*/(1 + [radiolabeled ligand]/K*_*d*_), where *IC*_*50 *_is the concentration of unlabeled ligand that produces 50% inhibition of the specific binding of radiolabeled ligand. The binding assay results are expressed as the mean ± SEM of four independent experiments, each performed in duplicate.

### cAMP assay

3',5'-Cyclic adenosine monophosphate (cAMP) assays were performed as previously described [17] with slight modifications. Briefly, 10^5 ^cells were placed into each well of a 24-well plate, grown for 24 h, washed, and incubated with 0.45 ml HEPES-buffered saline that contained 1 mM 3-isobutyl-1-methylxanthine for 10 min at 37°C. The cells were then stimulated for 10 min by the addition of 50 ml HEPES-buffered saline that contained 100 mM forskolin and 1 mM 3-isobutyl-1-methylxanthine in the presence or absence of various concentrations of opioid ligands and then disrupted by adding 0.5 ml ice-cold 10% trichloroacetic acid to each well. The concentrations of cAMP were measured by radioimmunoassay (Amersham, Buckinghamshire, UK). cAMP accumulation is expressed as a fraction of the control value obtained without the addition of opioids. Inhibition curves were generated using non-linear least-squares fit using GraphPad Prism (GraphPad, San Diego, CA). *IC*_*50 *_values were calculated as the concentration of ligand that produces 50% of maximal inhibition of cAMP accumulation. The *IC*_*50 *_values and maximal inhibitory effects (*I*_*max*_) in the cAMP assays are expressed as mean ± SEM of four independent experiments, each performed in triplicate.

### Statistical analyses

The dose-response functions of the thermal and mechanical antinociceptive effects of (-)-pentazocine were statistically evaluated by three-way, mixed-design analysis of variance (ANOVA) with two between-subjects factors (sex and genotype) and one within-subjects factor (drug dose). Differences among genotypes were statistically evaluated by two-way, mixed-design ANOVA followed by the Bonferroni *post hoc *test. The visceral chemical antinociceptive effects of (-)-pentazocine were analyzed by one-way and two-way factorial ANOVA followed by the Bonferroni *post hoc *test. The somatic chemical antinociceptive effects of (-)-pentazocine were statistically evaluated by four-way, mixed-design ANOVA with three between-subjects factors (drug treatment, sex, and genotype) and one within-subjects factor (time). The sum of the nociceptive scores during the 1st (0-15 min) and 2nd (15-60 min) phases were statistically evaluated by one-way factorial ANOVA followed by the Bonferroni *post hoc *test. The sum of the nociceptive scores of the (-)-pentazocine-treated groups were also analyzed by two-way factorial ANOVA with two between-subjects factors (genotype and sex). Values of *p *< 0.05 were considered statistically significant.

## Results

### Thermal antinociceptive effects

**Figure 1:**
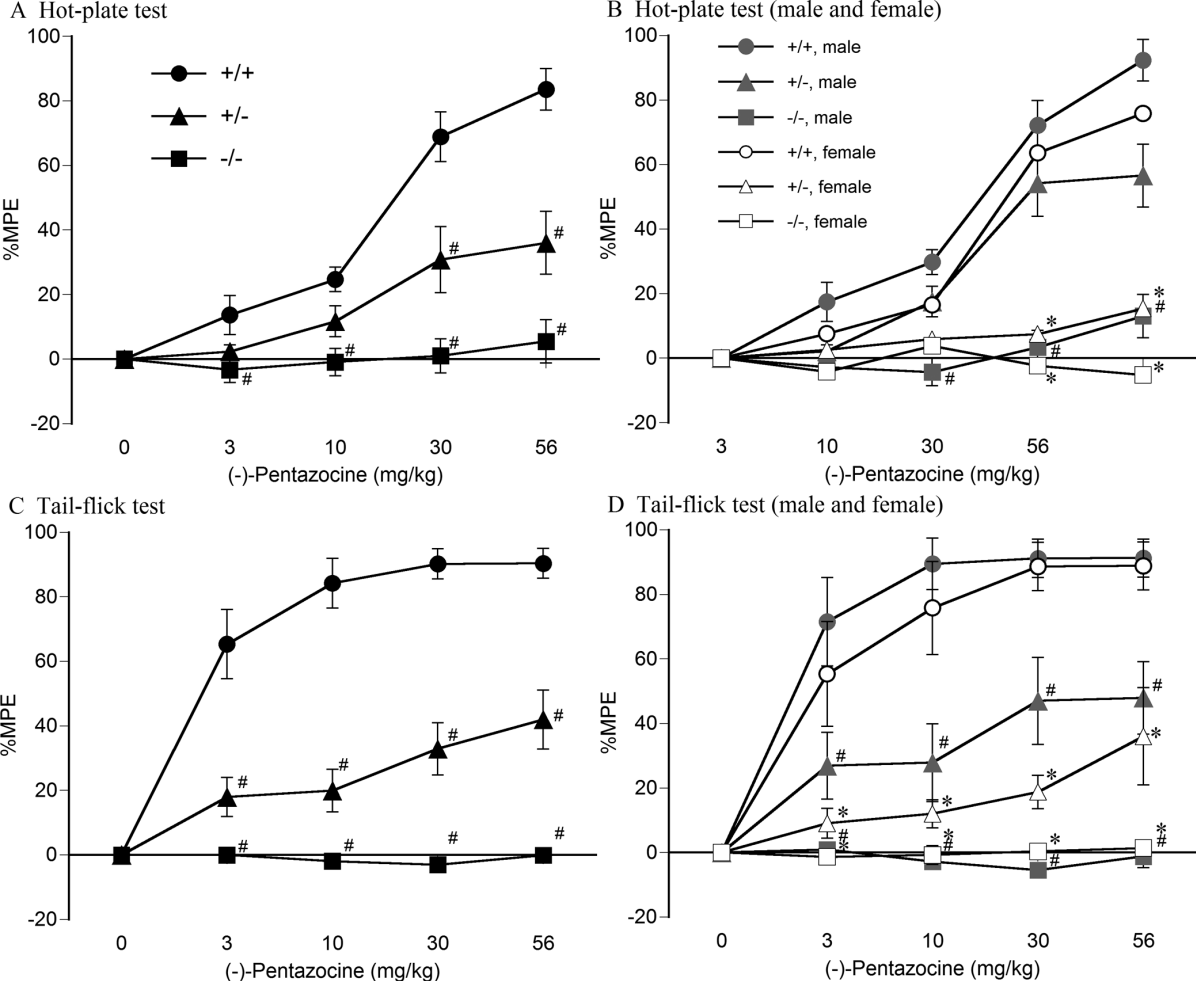
**Thermal antinociceptive effects of (-)-pentazocine in wildtype, heterozygous, and homozygous MOP-KO mice**. (-)-Pentazocine-induced alterations of %MPE in the hot-plate (A, B) and tail-flick (C, D) tests in wildtype (+/+; [A, C] closed circles, *n *= 12; [B, D] gray closed circles [male, *n *= 6], open circles [female, *n *= 6]), heterozygous (+/-; [A, C] closed triangles, *n *= 12; [B, D] gray closed triangles [male, *n *= 7], open triangles [female, *n *= 5]), and homozygous (-/-; [A, C] closed squares, *n *= 13; [B, D] gray closed squares [male, *n *= 8], open squares [female, *n *= 5]) MOP-KO mice under a cumulative dose-response paradigm. (A, C) Combined data of male and female mice. ^#^*p *< 0.05, significantly different from wildtype mice. (B, D) ^#^*p *< 0.05, significantly different from male wildtype mice; *p < 0.05, significantly different from female wildtype mice. Data are expressed as mean ± SEM.

The thermal antinociceptive dose-response relationships of (-)-pentazocine were analyzed in wildtype, heterozygous, and homozygous MOP-KO mice (Figure 1). (-)-Pentazocine dose-dependently induced thermal antinociceptive effects in both wildtype and heterozygous MOP-KO mice but not in homozygous MOP-KO mice. Three-way, mixed-design ANOVA revealed that the thermal antinociceptive effects of (-)-pentazocine (%MPE) were significantly different among these genotypes in both the hot-plate test (significant difference between genotypes, *F*_2,31 _= 34.39, *p *< 0.001; significant genotype × dose interaction, *F*_8,124 _= 13.53, *p *< 0.001; Figure 1A, B) and tail-flick test (significant difference between genotypes, *F*_2,31 _= 76.84, *p *< 0.001; significant genotype × dose interaction, *F*_8,124 _= 18.34, *p *< 0.001; Figure 1C, D). The thermal antinociceptive effects of (-)-pentazocine were significantly different between male and female mice in the hot-plate test (significant difference between sexes, *F*_1,31 _= 8.82, *p *< 0.01; significant sex × dose interaction, *F*_4,124 _= 4.16, *p *< 0.01; Figure 1B) but not in the tail-flick test (no significant difference between sexes, *F*_1,31 _= 2.30, *p *= 0.14; no sex × dose interaction, *F*_4,124 _= 0.78, *p *= 0.54; Figure 1D). Although the thermal antinociceptive effects of (-)-pentazocine in the tail-flick test tended to be more pronounced in male mice than in female mice, these differences were significant only in the hot-plate test.

In the hot-plate test, two-way, mixed-design ANOVA revealed that the thermal antinociceptive effects of (-)-pentazocine were significantly different among genotypes in both males (*F*_2,18 _= 18.23, *p *< 0.001) and females (*F*_2,13 _= 27.05, *p *< 0.001; Figure 1B). The thermal antinociceptive effects of (-)-pentazocine in heterozygous and homozygous MOP-KO female mice were significantly lower than in wildtype female mice (*p *< 0.05, Bonferroni *post hoc *test). By contrast, these effects only in homozygous MOP-KO male mice were significantly lower than in wildtype male mice (*p *< 0.05, Bonferroni *post hoc *test). Furthermore, two-way, mixed-design ANOVA also revealed that the thermal antinociceptive effects of (-)-pentazocine were significantly different between male and female heterozygous MOP-KO mice (*F*_1,10 _= 8.31, *p *< 0.05) but not in wildtype and homozygous MOP-KO mice.

In the tail-flick test, two-way, mixed-design ANOVA revealed that the thermal antinociceptive effects of (-)-pentazocine were significantly different among genotypes (*F*_2,34 _= 78.85, *p *< 0.001; Figure 1C, D). The thermal antinociceptive effects of (-)-pentazocine in heterozygous and homozygous MOP-KO mice were significantly lower than in wildtype mice (*p *< 0.05, Bonferroni *post hoc *test).

### Mechanical antinociceptive effects

**Figure 2:**
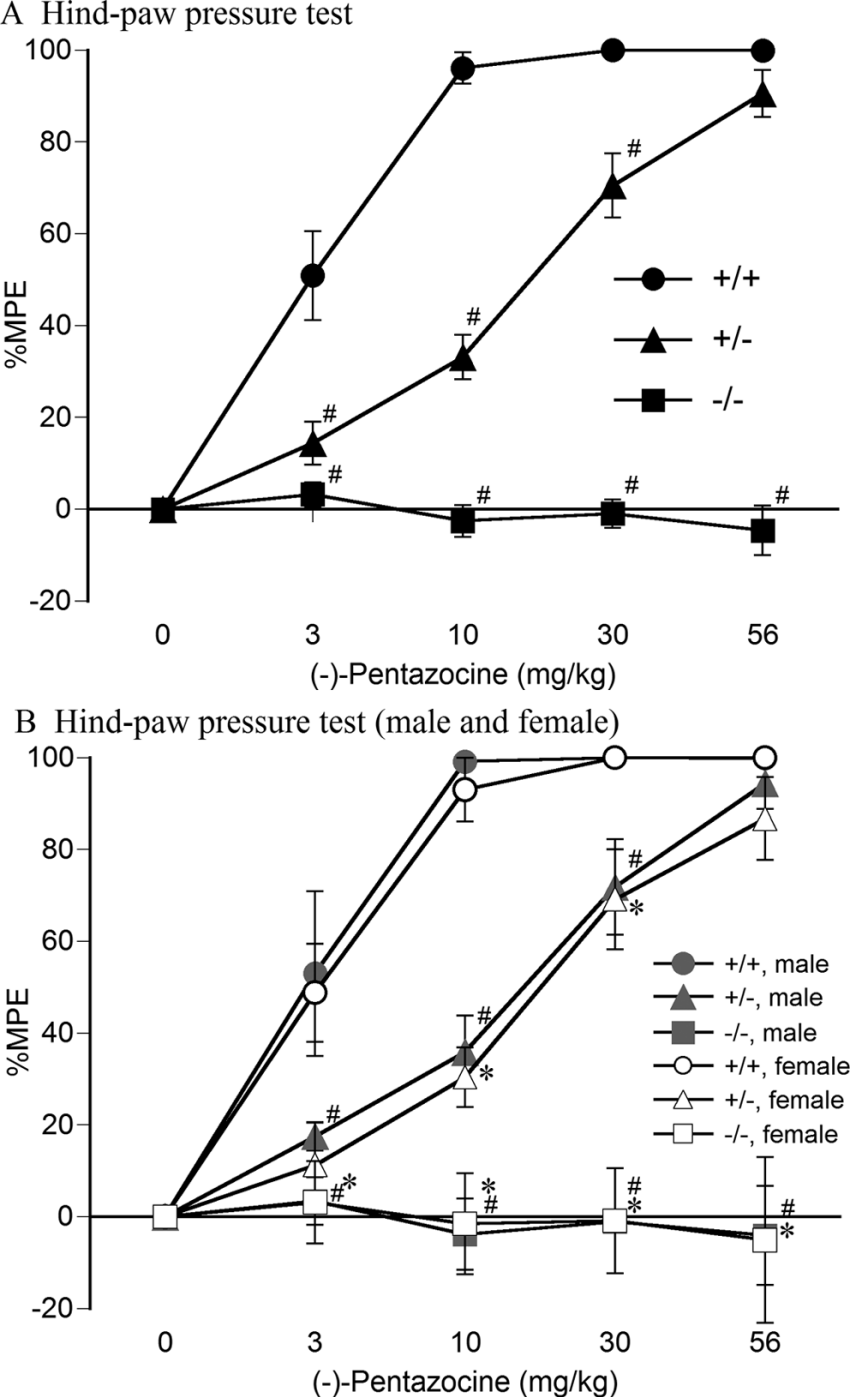
**Mechanical antinociceptive effects of (-)-pentazocine in wildtype, heterozygous, and homozygous MOP-KO mice**. (-)-Pentazocine-induced alterations of %MPE in the hind-paw pressure test in wildtype (+/+; [A] closed circles, *n *= 8; [B] gray closed circles [male, *n *= 4], open circles [female, *n *= 4]), heterozygous (+/-; [A] closed triangles, *n *= 9; [B] gray closed triangles [male, *n *= 4], open triangles [female, *n *= 5]), and homozygous (-/-; [A] closed squares, *n *= 8; [B] gray closed squares [male, *n *= 4], open squares [female, *n *= 4]) MOP-KO mice under a cumulative dose-response paradigm. (A) Combined data of male and female mice. ^#^*p *< 0.05, significantly different from wildtype mice. (B) ^#^*p *< 0.05, significantly different from male wildtype mice; *p < 0.05, significantly different from female wildtype mice. Data are expressed as mean ± SEM.

The mechanical antinociceptive effects of (-)-pentazocine were then analyzed in wildtype, heterozygous, and homozygous MOP-KO mice (Figure 2). (-)-Pentazocine showed dose-dependent mechanical antinociceptive effects in both wildtype and heterozygous MOP-KO mice but not in homozygous MOP-KO mice. Three-way, mixed-design ANOVA revealed that the mechanical antinociceptive effects of (-)-pentazocine were significantly different among these genotypes in the hind-paw pressure test (significant difference between genotypes, *F*_2,19 _= 233.2, *p *< 0.001; significant genotype × dose interaction, *F*_8,76 _= 38.29, *p *< 0.001; Figure 2). In contrast, these effects in the hind-paw pressure test were not significantly different between male and female mice (no significant difference between sexes, *F*_1,19 _= 0.58, *p *= 0.45; no sex × dose interaction, *F*_4,124 _= 0.78, *p *= 0.54; Figure 2B). Two-way, mixed-design ANOVA revealed that the mechanical antinociceptive effects of (-)-pentazocine were significantly different among genotypes (*F*_2,22 _= 257.5, *p *< 0.001; Figure 2A). The mechanical antinociceptive effects of (-)-pentazocine in both heterozygous and homozygous MOP-KO mice were significantly lower than in wildtype mice (*p *< 0.05, Bonferroni *post hoc *test).

### Visceral chemical antinociceptive effects

**Figure 3:**
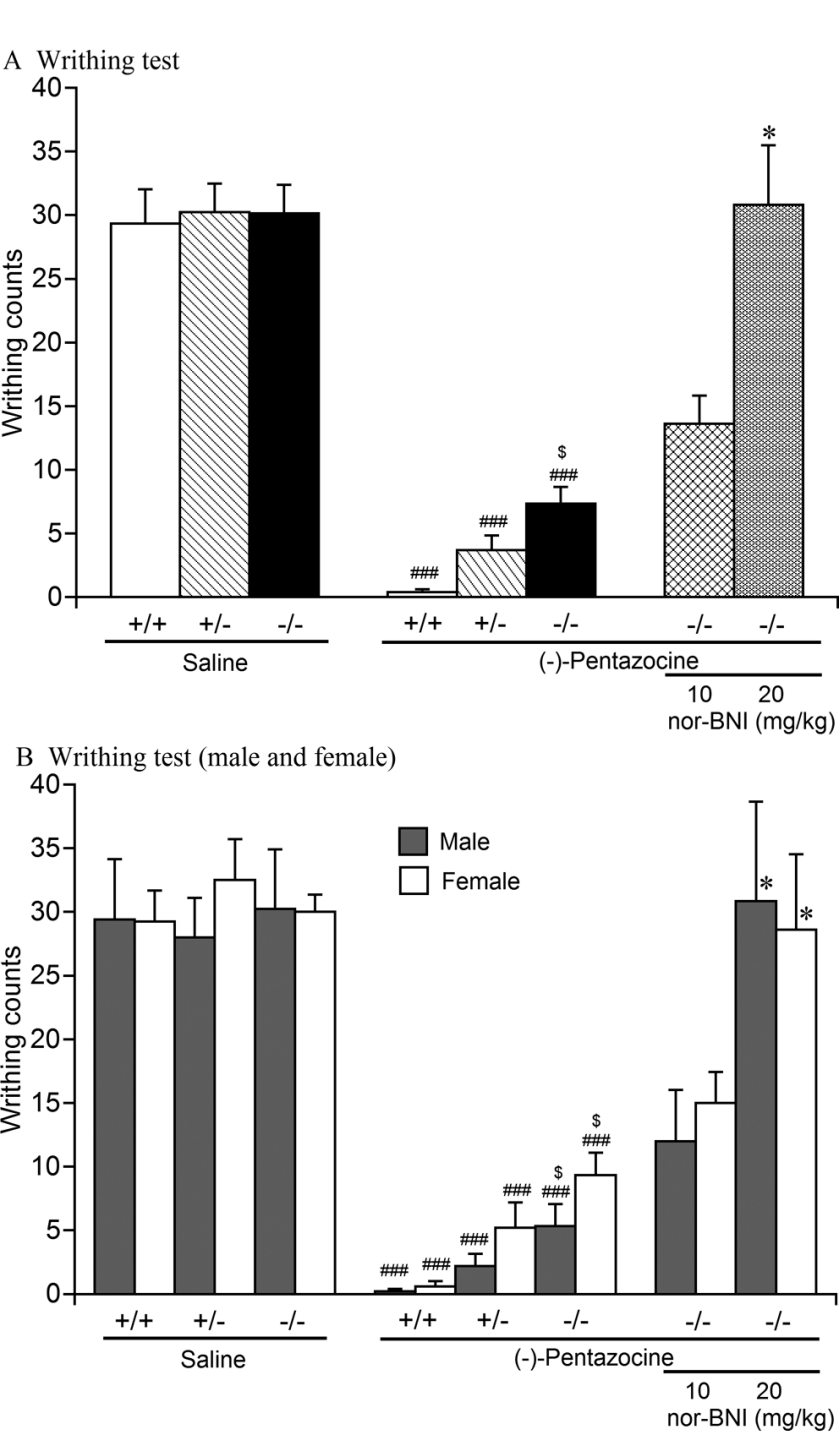
**Visceral chemical antinociceptive effects of (-)-pentazocine in wildtype, heterozygous, and homozygous MOP-KO mice**. Writhing counts induced by 0.6% acetic acid (i.p.) with saline pretreatment in wildtype (+/+; [A] *n *= 9; [B] male [*n *= 5], female [*n *= 4]), heterozygous (+/-; [A] *n *= 8; [B] male [*n *= 8], female [*n *= 4]), and homozygous (-/-; [A] *n *= 8; [B] male [*n *= 4], female [*n *= 4]) mice, (-)-pentazocine pretreatment (10 mg/kg, s.c.) in wildtype (+/+; [A] *n *= 10; [B] male [*n *= 5], female [*n *= 5]), heterozygous (+/-; [A] *n *= 10; [B] male [*n *= 5], female [*n *= 5]), and homozygous (-/-; [A] *n *= 12; [B] male [*n *= 6], female [*n *= 6]) MOP-KO mice, and nor-BNI (10, 20 mg/kg, s.c.) and (-)-pentazocine (10 mg/kg, s.c.) pretreatment (10 mg/kg nor-BNI: [A] *n *= 11; [B] male [*n *= 5], female [*n *= 6]; 20 mg/kg nor-BNI: [A] *n *= 11; [B] male [*n *= 6], female [*n *= 5]) in homozygous MOP-KO mice. (A) Combined data of male and female mice. (A, B) ^###^*p *< 0.001, significantly different from saline pretreatment; ^$^*p *< 0.05, significantly different from wildtype mice. *p < 0.05, significantly different from (-)-pentazocine-pretreated homozygous MOP-KO mice. Data are expressed as mean ± SEM.

The visceral chemical antinociceptive effects of (-)-pentazocine (10 mg/kg, s.c.) were analyzed in wildtype, heterozygous, and homozygous MOP-KO mice using the writhing test. Interestingly, (-)-pentazocine induced visceral chemical antinociceptive effects not only in wildtype and heterozygous MOP-KO mice, but also in homozygous MOP-KO mice. One-way factorial ANOVA revealed that (-)-pentazocine significantly decreased writhing (Figure 3A) in wildtype mice (*F*_1,17 _= 128.1, *p *< 0.001), heterozygous MOP-KO mice (*F*_1,16 _= 125.4, *p *< 0.001), and homozygous MOP-KO mice (*F*_1,18 _= 87.40, *p *< 0.001). Although no significant differences in writhing counts were observed in the saline-treated groups, two-way factorial ANOVA with two between-subjects factors (genotype and sex) showed significant differences in writhing counts between genotypes in the (-)-pentazocine-treated group (*F*_2,26 _= 12.06, *p *< 0.001). Furthermore, writhing counts in female mice in the (-)-pentazocine-treated group were higher than in male mice (*F*_1,26 _= 4.42, *p *< 0.05; Figure 3B). Writhing counts in (-)-pentazocine-treated homozygous MOP-KO mice were significantly higher than in both male and female wildtype mice treated with (-)-pentazocine (*p *< 0.05, Bonferroni *post hoc *test).

The remaining visceral chemical antinociceptive effects of (-)-pentazocine in homozygous MOP-KO mice were dose-dependently antagonized by pretreatment with nor-BNI (s.c.). Two-way factorial ANOVA with two between-subjects factors (sex and nor-BNI dose) in MOP-KO mice revealed a significant difference in writhing counts between nor-BNI doses (*F*_2,28 _= 48.07, *p *< 0.05) but no difference between males and females. Treatment with 20 mg/kg nor-BNI in MOP-KO mice significantly antagonized the remaining visceral chemical antinociceptive effects of (-)-pentazocine (*p *< 0.05, Bonferroni *post hoc *test).

### Somatic chemical antinociceptive effects

**Figure 4:**
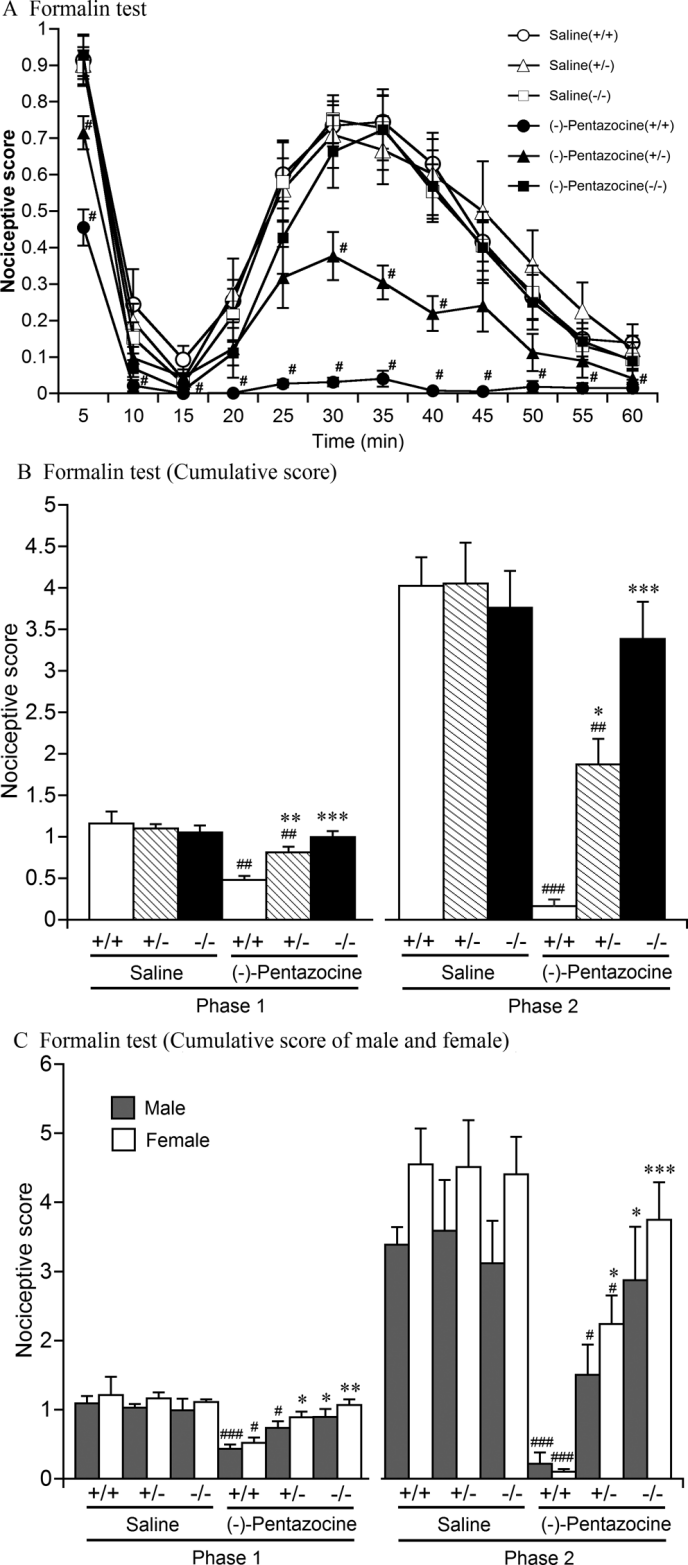
**Somatic chemical antinociceptive effects of (-)-pentazocine in wildtype, heterozygous, and homozygous MOP-KO mice**. Time-course of (A) and cumulative (B, C) nociceptive scores of formalin-induced nociceptive behavior with saline pretreatment in wildtype (+/+; male [*n *= 5], female [*n *= 6]), heterozygous (+/-; male [*n *= 5], female [*n *= 5]), and homozygous (-/-; male [*n *= 5], female [*n *= 5]) mice and (-)-pentazocine pretreatment (10 mg/kg, s.c.) in wildtype (+/+; male [*n *= 5], female [*n *= 5]), heterozygous (+/-; male [*n *= 5], female [*n *= 5]), and homozygous (-/-; male [*n *= 5], female [*n *= 7]) MOP-KO mice. (A, B) Combined data of male and female mice. (A-C) ^#^*p *< 0.05, ^##^*p *< 0.005, ^###^*p *< 0.001, significantly different from saline pretreatment. (B, C) *p < 0.05, **p < 0.005, ***p < 0.001, significantly different from wildtype mice. Data are expressed as mean ± SEM.

The somatic chemical antinociceptive effects of (-)-pentazocine (10 mg/kg, s.c.) were analyzed in wildtype, heterozygous, and homozygous MOP-KO mice using the formalin test. (-)-Pentazocine exerted somatic chemical antinociceptive effects in both wildtype and heterozygous MOP-KO mice but not in homozygous MOP-KO mice (Figure 4). Four-way, mixed-design ANOVA with three between-subjects factors (sex, genotype, and drug treatment) and one within-subjects factor (time) revealed that the nociceptive scores in the formalin test were significantly different among these genotypes (significant difference between genotypes, *F*_2,51 _= 8.26, *p *< 0.005; significant genotype × drug treatment interaction, *F*_2,51 _= 11.45, *p *< 0.001; significant genotype × time interaction, *F*_22,561 _= 2.82, *p *< 0.001; significant genotype × time × drug treatment interaction, *F*_22,561 _= 2.20, *p *< 0.005; Figure 4A). Moreover, we found a significant difference between sexes (*F*_1,51 _= 7.57, *p *< 0.01) and a significant sex × time interaction (*F*_11,561 _= 2.97, *p *< 0.005) but no sex × drug treatment interaction.

Two phases of spontaneous nociceptive behavior were analyzed (phase 1 beginning at 0 min and lasting for 15 min, and phase 2 beginning at 15 min). Therefore, the effects of (-)-pentazocine were based on the cumulative number of nociceptive scores for each phase for each mouse (Figure 4B, C). One-way factorial ANOVA revealed that (-)-pentazocine significantly decreased nociceptive scores during both phases (Figure 4B) in wildtype mice (Phase 1, *F*_1,19 _= 16.79, *p *< 0.005; Phase 2, *F*_1,19 _= 99.92, *p *< 0.001) and heterozygous MOP-KO mice (Phase 1, *F*_1,18 _= 11.46, *p *< 0.005; Phase 2, *F*_1,18 _= 13.97, *p *< 0.005) but not in homozygous MOP-KO mice. Although no significant differences in nociceptive scores were observed in the saline-treated groups, two-way factorial ANOVA with two between-subjects factors (genotype and sex) showed significant differences in nociceptive scores between the genotypes in the (-)-pentazocine-treated groups (Phase 1, *F*_2,26 _= 16.33, *p *< 0.001; Phase 2, *F*_2,26 _= 21.81, *p *< 0.001). The nociceptive scores of both the heterozygous and homozygous MOP-KO mice in the (-)-pentazocine-treated groups were significantly higher than in wildtype mice during both phases (*p *< 0.05, Bonferroni *post hoc *test). By contrast, although the nociceptive scores of female mice in the (-)-pentazocine-treated groups tended to be higher than those of male mice, no significant differences were observed between sexes (Phase 1, *F*_1,26 _= 3.50, *p *= 0.073; Phase 2, *F*_1,26 _= 1.59, *p *= 0.219; Figure 4C).

### Binding characteristics

**Figure 5:**
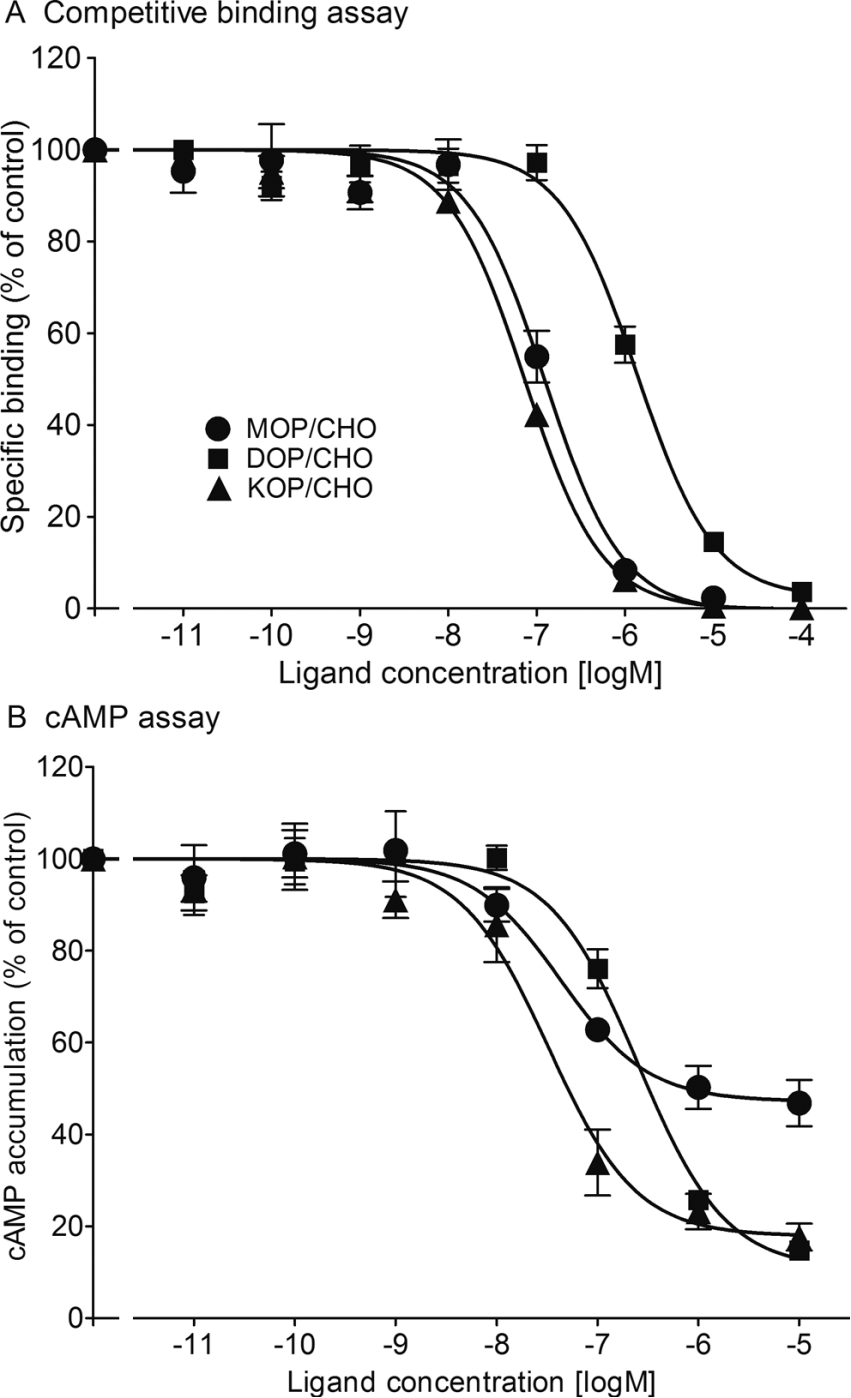
**Binding properties and agonist effects of (-)-pentazocine on human opioid receptor subtypes**. (A) Binding properties of (-)-pentazocine for displacement of specific binding of 2 nM [^3^H]DAMGO, 2 nM [^3^H]DPDPE, and 3 nM [^3^H]U69593 to membranes of MOP/CHO cells (closed circles, *n *= 4), DOP/CHO cells (closed squares, *n *= 4), and KOP/CHO cells (closed triangles, *n *= 4), respectively. Data are expressed as mean ± SEM. (B) Agonist effects of (-)-pentazocine on forskolin-stimulated cAMP production in MOP/CHO cells (closed circles, *n *= 4), DOP/CHO cells (closed squares, *n *= 4), and KOP/CHO cells (closed triangles, *n *= 4). Intracellular cAMP levels in the cells incubated with 10 mM forskolin alone served as controls (100%). Data are expressed as mean ± SEM.

(-)-Pentazocine competition experiments using membranes prepared from MOP/CHO, DOP/CHO, and KOP/CHO cells revealed apparent binding affinities for each opioid receptor subtype (Figure 5A, Table 1). (-)-Pentazocine bound with higher affinity than morphine to membranes prepared from KOP/CHO cells. The morphine results were obtained from previous data [17] that were reanalyzed according to the present methods. Although the affinity of (-)-pentazocine for the KOP receptor was slightly higher than for the MOP receptor, (-)-pentazocine showed moderate affinity for the MOP receptor. The affinities of (-)-pentazocine for MOP and KOP receptors were higher than for DOP receptors.

**Table 1:** Binding properties and agonist effects of (-)-pentazocine and morphine on human opioid receptor subtypes

		**MOP/CHO**	**DOP/CHO**	**KOP/CHO**
**Competitive binding assay**				

K_i _value (nM)				

(-)-Pentazocine		85.6 ± 13.3	641 ± 88	35.2 ± 2.6

Morphine		21.0 ± 3.7	524 ± 83	247 ± 13

**cAMP assay**				

IC_50 _(nM)				

(-)-Pentazocine		42.8 ± 12.9	255 ± 46	39.6 ± 14.8

Morphine		25.0 ± 9.0	610 ± 220	340 ± 160

I_max _(%)				

(-)-Pentazocine		52.8 ± 3.0	89.3 ± 4.3	82.1 ± 3.7

Morphine		88.0 ± 3.1	83.7 ± 2.7	84.3 ± 3.3

Morphine data were obtained from a reanalysis of the data in [10].

### cAMP assay

The effects of (-)-pentazocine on forskolin-stimulated cAMP accumulation in MOP/CHO, DOP/CHO, and KOP/CHO cells were also tested. (-)-Pentazocine concentration-dependently suppressed forskolin-stimulated cAMP accumulation in all three cell types (Figure 5B). The *I*_*max *_values of (-)-pentazocine were lower than those of morphine for MOP/CHO cells and were the same as those of morphine for DOP/CHO and KOP/CHO cells (Table 1). The *IC*_*50 *_values of (-)-pentazocine were lower than those of morphine for DOP/CHO and KOP/CHO cells (Table 1). The morphine results were obtained from previous data [17] that were reanalyzed according to the present methods. The *IC*_*50 *_values of (-)-pentazocine for MOP/CHO cells were nearly the same as those for KOP/CHO cells.

## Discussion

In the present study, the antinociceptive effects of (-)-pentazocine on various types of nociceptive stimuli were significantly reduced in heterozygous and homozygous MOP-KO mice compared with wildtype mice. The antinociceptive effects of (-)-pentazocine in these tests increased in a MOP receptor gene dose-dependent fashion. The copy numbers of the MOP receptor gene are zero in homozygous MOP-KO mice, one in heterozygous MOP-KO mice, and two in wildtype mice. These results were obtained in not only male but also female mice, although female mice may respond differently in pain tests during different phases of their estrous cycle [18]. These results suggest that the MOP receptor is the main opioid receptor involved in (-)-pentazocine-induced antinociception. The antinociceptive effects of (-)-pentazocine were previously hypothesized to be mediated by its agonist action at KOP receptors [13]. A previous report showed that the antinociceptive effects of (-)-pentazocine were antagonized by nor-BNI, a selective KOP receptor antagonist, but not by β-funaltrexamine, a selective MOP receptor antagonist, in the mouse tail-flick test [3]. However, other groups reported that the antinociceptive effects of (-)-pentazocine were antagonized by β-funaltrexamine in the mouse hot-plate test [3] and writhing test [4]. The discrepancy between these studies might be attributable to differences in the type of nociceptive test, strain of mice, or injection route. Furthermore, the most selective ligands for a specific subtype of opioid receptors possess certain affinities for other opioid receptor subtypes [5]. Some *in vivo *studies also demonstrated antagonist effects of nor-BNI at other opioid receptor subtypes [19,20]. Thus, the role of the MOP receptor in the antinociceptive effects of (-)-pentazocine has not been clearly evaluated by traditional pharmacological studies that only used selective ligands. The results of our *in vitro *experiments that used human MOP, DOP, and KOP receptor cDNA suggest that (-)-pentazocine induces its antinociceptive effects via the MOP receptor in humans. Although (-)-pentazocine bound to human MOP receptor with moderate affinity and showed moderate *I*_*max *_values for the MOP receptor in the cAMP assays, (-)-pentazocine had high *IC*_*50 *_values for the MOP receptor in the cAMP assays, which were nearly the same as the *IC*_*50 *_values for the KOP receptor and the *IC*_*50 *_values of morphine for the MOP receptor. These results suggest that the MOP receptor could be involved in the antinociceptive effects of (-)-pentazocine in humans and rodents.

The antinociceptive effects of morphine, a MOP receptor agonist with low affinity for DOP and KOP receptors, are reduced in several strains of heterozygous MOP-KO mice and completely abolished in homozygous MOP-KO mice [7-9]. Furthermore, the thermal and mechanical antinociception induced by buprenorphine and butorphanol, nonselective opioid receptor partial agonists, are abolished in MOP-KO mice [10,11]. In contrast, the antinociceptive effects of morphine are not altered in mice that lack the DOP receptor [21] or in mice that lack the KOP receptor [22]. The present results, together with these previous reports, suggest that the MOP receptor may play a critical role in the analgesia induced by opioid partial agonists. MOP receptor tolerance and inactivation or individual differences in the number of MOP receptors are thus important for most of the variations in the degree of analgesia induced by opioids. Still unclear, however, is whether DOP and KOP receptors modulate the antinociceptive effects of not only (-)-pentazocine but also other opioid partial agonists. Further studies of DOP-KO, KOP-KO, and double DOP/KOP-KO mice will reveal the mechanisms that underlie these antinociceptive effects.

In contrast to thermal, mechanical, and somatic chemical antinociception, (-)-pentazocine exerted significant visceral chemical antinociception in homozygous MOP-KO mice, although the visceral chemical antinociceptive effects of (-)-pentazocine increased in a MOP receptor gene dose-dependent fashion. The residual visceral chemical antinociception induced by (-)-pentazocine was abolished by pretreatment with nor-BNI. These results indicate that both MOP and KOP receptors play dominant roles in (-)-pentazocine-induced visceral chemical antinociception, which is consistent with previous reports. The enhanced response of KOP-KO mice in the acetic acid writhing test has been previously demonstrated [22]. Furthermore, butorphanol has been shown to abolish thermal and mechanical antinociception and the nor-BNI-sensitive retention of visceral chemical antinociception in MOP-KO mice [11]. The present results, together with previous studies, suggest that both MOP and KOP receptors play important roles in visceral chemical analgesia mediated by opioid partial agonists. Furthermore, both MOP and KOP receptor-selective agonists reportedly exert significant antinociceptive effects in mice in a visceral mechanical pain model that utilizes colorectal distension [23], and peripheral KOP receptor agonists reportedly reduce visceral pain in humans [24]. The pain pathways that mediate visceral and somatic pain have several differences [25]. Notably, treatments with KOP but not MOP or DOP receptor agonists have been shown to attenuate the responses of afferent fibers to colorectal distension [26]. The KOP receptor may play a primary role in the antinociceptive effect of opioid agonists on visceral pain via peripheral mechanisms, and MOP and KOP receptors may play a role via central mechanisms. The present results, together with previous studies, suggest that pain induced by various visceral stimuli can be better controlled by a nonselective opioid that acts at both MOP and KOP receptors. Further studies on the receptor mechanisms that underlie the analgesic effects of opioids will lead to the development of better clinical treatments of various types of pain.

Sex differences in the antinociceptive effects of (-)-pentazocine were also demonstrated in the present study. The antinociceptive effects of (-)-pentazocine were significantly higher in male than in female mice in both the hot-plate and writhing tests and tended to be high in the tail-flick and formalin tests. These sex differences appear to be pronounced in heterozygous MOP-KO mice, although sex differences in the antinociceptive effects of (-)-pentazocine in wildtype mice might not be noticeable because of a possible ceiling effect in the present nociceptive tests. The present results are consistent with previous reports. Pentazocine has been shown to exert more potent antinociception in males than in females in both mice [27] and rats [28]. These reports also showed that U50488H, a selective KOP receptor agonist, and other opioids (e.g., U69593, bremazocine, and butorphanol) are more effective in males than in females. Furthermore, with regard to MOP receptor-selective agonists, morphine exerted greater antinociceptive effects in male than in female mice [29], rats [30,31], and monkeys [32]. Additionally, female mice may differentially respond in pain tests during different phases of their estrous cycle [18]. In humans, males required less morphine or fentanyl than females for postoperative pain relief [33,34]. In contrast, some inconsistent human studies have reported that the antinociceptive effects of pentazocine on postoperative pain were higher in females than in males [35-37]. The discrepancy between these studies might be attributable to differences in the body weight-adjusted dose of pentazocine, although other factors (e.g., type of nociceptive stimulus, type of clinical surgery, estrous cycle phase, patient race, and ethnicity) might affect these results. Thus, the present results, together with previous reports, suggest that not only MOP receptor-selective opioids, but also other subtype-nonselective opioids such as pentazocine, are more effective in males than in females.

## Conclusions

The present study demonstrated the abolition of the thermal, mechanical, and somatic chemical antinociceptive effects of (-)-pentazocine in male and female MOP-KO mice, suggesting that thermal, mechanical, and somatic chemical antinociception induced by (-)-pentazocine is completely mediated by the MOP receptor partial agonist effects of (-)-pentazocine. We also demonstrated the retention of (-)-pentazocine-induced visceral chemical antinociception in MOP-KO mice and abolition of (-)-pentazocine-induced visceral chemical antinociception by pretreatment with nor-BNI. Our *in vitro *data showed that (-)-pentazocine more strongly acted at KOP and MOP receptors than DOP receptors, suggesting that (-)-pentazocine-induced visceral chemical antinociception is mediated by its MOP receptor partial agonist effects and full KOP receptor agonist effects. In the clinic, (-)-pentazocine may effectively control visceral pain. Future studies will elucidate the precise molecular mechanisms that underlie the antinociceptive effects of (-)-pentazocine and will contribute to the better use of opioid drugs for pain management.

## Authors' contributions

The study was conceived and the experiments were designed by SI, MM, MS, and KI. SI performed the experiments, performed the statistical analyses, and wrote the manuscript. MOP-KO mice were developed by IS and GRU. KI supervised the experiments and finalized the manuscript. All authors contributed to writing the manuscript, and all authors read and approved the final manuscript.

## Competing interests

The authors declare that they have no competing interests.
